# Preliminary validation of a novel tool to assess dog welfare: The Animal Welfare Assessment Grid

**DOI:** 10.3389/fvets.2022.940017

**Published:** 2022-09-16

**Authors:** Rachel Malkani, Sharmini Paramasivam, Sarah Wolfensohn

**Affiliations:** School of Veterinary Medicine, University of Surrey, Guildford, United Kingdom

**Keywords:** dog, welfare assessment, quality of life, validation, veterinary medicine

## Abstract

Animal welfare monitoring is a vital part of veterinary medicine and can be challenging due to a range of factors that contribute to the perception of welfare. Tools can be used, however; there are few validated and objective methods available for veterinary and animal welfare professionals to assess and monitor the welfare of dogs over their lifetime. This study aimed to adapt a framework previously validated for other species, The Animal Welfare Assessment Grid (AWAG), for dogs and to host the tool on an accessible, easy to use online platform. Development of the AWAG for dogs involved using the scientific literature to decide which factors were relevant to score welfare in dogs and to also write the factor descriptors. The primary tool was trialed with veterinary professionals to refine and improve the AWAG. Content validity was assessed by subject matter experts by rating the validity of the factors for assessing dog welfare using the item-level content validity index (I-CVI) and scale-level content validity index based on the average method (S-CVI/Ave). Construct validity was evaluated by users of the tool scoring healthy and sick dogs, as well as healthy dogs undergoing neutering procedures. Mann Whitney tests demonstrate that the tool can differentiate between healthy and sick dogs, and healthy and healthy dogs post elective surgery. Test re-test reliability was tested by users conducting multiple assessments on individual dogs under non-changing conditions. Inter-rater reliability was assessed by two users scoring an individual dog at the same time in veterinary referral practice. Repeated measures ANOVA for test re-test and inter-rater reliability both show no statistical difference between scores and that the scores are highly correlated. This study provides evidence that the AWAG for dogs has good content and construct validity, alongside good test re-test and inter-rater reliability.

## Introduction

There is currently no universally accepted method to assess animal welfare in any species; however multiple indicators that are evidenced to impact wellbeing both positively and negatively should be used to evaluate an animal's welfare state (1). Routine welfare assessment often needs to produce rapid results, be non-invasive, and should not require any special training for ease of use (2). Assessment tools that are used to evaluate welfare must be developed to be as objective as possible and this can be achieved by ensuring the tool is valid and reliable (3).

Producing reliable results means that similar results occur each time the same animal is tested under the same conditions. Inconsistent results may lead to an inaccurate assessment of welfare and weaken the strength of research findings. It is important to include multiple measures of reliability testing to ensure the tool is consistently reliable by using methods such as inter-rater reliability and test re-testing reliability (Table 1).

**Table 1:** Measures of reliability.

**Measures of reliability**	
Test-retest reliability	Ability of the measuring tool to reproduce the same results consistently over time
Internal consistency reliability	Consistency of results across associated items within a tool
Interrater reliability	Multiple users achieve similar results when scoring at the same time point

The validity of a tool means that it is accurately measuring the construct that the tool was designed to measure. Various tests of validity should be used to establish the tool measures an animal's welfare state and these tests may include construct and content validity (Table 2).

**Table 2:** Measures of validity.

**Measures of validity**	
Content validity	A review by an expert panel to decide whether the tool represents the construct you are measuring
Construct validity	Assessing the ability to evaluate and discriminate between different constructs

In order to get a true picture of an animal's quality of life, welfare should be routinely measured and not just taken at a snapshot in time. Most of the discussions relating to quality of life in the veterinary clinical setting use no objective tools to assess the animal's welfare. These discussions are more often prompted by owners than vets and may center mainly around euthanasia decisions rather than the proactive quality of life improvement initiatives at an earlier stage (4). Achieving patient-centric and welfare-based treatment goals throughout the animal's life often depends on the willingness of the client and there is an increasing amount of research on improving communication skills and how to implement change in pet owners (5). However, there are few tools for veterinary and animal welfare professionals to use that objectively and holistically assess dog welfare.

Tools that have been previously developed to assess quality of life largely relate to clinical health and illness. Health related quality of life assessment instruments typically take the form of structured questionnaires, which can be either generic or disease specific.

Disease specific instruments may be more responsive to clinical change, but generic instruments can be more valuable as they have the ability to assess a range of factors that impact welfare (6). Quality of life (QOL) tools that have been developed for various chronic diseases and illnesses include chronic pain (7), cardiac disease (8), spinal cord injury (9), osteoarthritis (10, 11), cancer (12, 13), and atopic dermatitis (14–16).

There have also been many tools developed to assess QOL that are unrelated to a specific disease. Mullan and Main (17) devised a four-part quality of life questionnaire for dog owners that assesses a variety of components that can impact a dog's welfare including pain, comfort, exercise, diet, mental stimulation, companionship with people and other dogs. The Mullan and Main (17) tool also assesses behavioral health which is often overlooked in other assessments. It also incorporates a simplified version of the health-related quality-of-life questionnaire for dogs with chronic pain (7). The strong merit of this tool is that it assesses the dog holistically and is patient-centered; it has good repeatability, internal consistency, and validity. However, the questionnaire was designed as a screening tool that would raise awareness of welfare considerations of dogs in veterinary practice, not to generate a quantifiable measure of welfare, and therefore cannot be used to compare the quality of life within or between dogs or measure change over time.

Schmutz et al. (18) developed a tool that is completed by dog owners and assesses eight parameters (energetic, mobile, relaxed, happy, sociable, relaxed, interested and satisfied) using a Likert-type scale. The instrument is demonstrated to have good content validity and reliability and poorer scores are shown in dogs that have chronic disease, demonstrating the tool's use to detect the negative impact of these conditions. The authors also state that the instrument can be completed quickly (3–5 min), which is an important aspect that users may consider when deciding whether to use a welfare assessment tool.

Reid et al. (19) previously designed and validated a similar instrument, the VetMetrica health-related quality of life (HRQL) assessment tool in which the dog owner completes 22 questions and the tool produces scores across four parameters of quality of life (energy, happiness, comfort and calmness). The user can then generate a summary score for physical wellbeing and emotional wellbeing. The user can also compare the scores of the four domains to the average healthy dog in the individuals age group. The scores can assess treatment success to measure clinically significant change over time. The VetMetrica HRQL tool, similar to the Mullan and Main questionnaire, takes a holistic approach to evaluate the dog's quality of life; however, Reid et al. additionally assess how the animal feels about its situation and compares this to what is the average healthy score for the dog's age and breed. However, there is likely to be variation between and within dog breeds and ages, so it may be difficult to compare a dog to another of the same breed or age. Rodger et al. (20) explored the variation of age, sex, and breed using the VetMetrica HRQL tool and found in three domains (energetic and enthusiastic, happy and content, and active and comfortable) there was variation with age, but HRQL declined as the dog aged. This finding is unsurprising as the prevalence of health problems in older animals increases with age. However, in general, there was found to be considerable variation in the HRQL scores, in particular, amongst breeds across all HRQL parameters.

Therefore, a QOL assessment tool should consider each dog as an individual with their own subjective emotions. Emotions can be defined as mental states that motivate behavior by facilitating adaptive physiological, cognitive and behavioral responses (21, 22). Although these cannot be measured directly, there are increasing studies investigating a range of emotional states in dogs (23–28), in particular negative affective states such as fear, anxiety, and frustration. Furthermore, brain anatomy associated with emotional processing in humans are similarly identified in dogs (29–33).

Belshaw (4) states that QOL assessment should encourage us to see each animal as an individual in how they are affected by illnesses and interventions, social interactions and changes in living conditions. Basing quality-of-life assessment on the individual patient and designing unique care and improvement has also been a recent development in human health care. Some studies in mental health have found many benefits to this approach as it allows the patients' needs and interventions to be planned around what they perceive to be important to their quality of life.

Although these instruments make a great effort to maintain objectivity, relying on owner reports alone may be problematic as their perception of their dog can influence their reporting. Owner interpretation of how well or poorly each dog is coping is going to be largely subjective, since it will depend on the individual, and potentially biased, perceptions and beliefs of whoever is making the judgment (34). For example, when assessing their dog's body condition, owners are reported to underestimate their dog's, despite using an objective body condition scale (35). Moreover, owner compliance in regularly completing a questionnaire may be difficult to obtain (36). Therefore, combining a structured owner report, clinical examination, observation of behavior, and discussion of the animal in question may give a better chance of an accurate insight into the dog's welfare. However, it is inevitable that a certain amount of subjectivity may remain in a QOL assessment, but using well-structured tools that aim to reduce bias should help to mitigate this.

It is rare that the instruments incorporate the past experiences of the dog, nor do they consider the actual impact that treatment may have, or predict prospective welfare. Most tools are made to capture the “in the moment” picture of the dog's welfare or look at trends of welfare scores over time.

A common approach for constructing QOL instruments is to identify various domains that independently impact welfare and this concept allows the consideration of the multiple components that reflect the multifactorial nature of quality of life (37). However, many factors that influence a dog's welfare are not independent; pain will affect the dog's behavior, and their ability to play and interact with people and other animals. A change in environment may alter their emotional state, and thus their ability to make choices or carry out behavior. Therefore, when assessing welfare, each factor is likely to influence other aspects of the dog's life, but it is still important to score each individual factor to ascertain where welfare can be improved.

More recently, the issue of novel or “heroic” treatments has raised concern over how QOL is assessed or considered when assessing treatment options. Therefore, objective, structured tools that help assess QOL and help make decisions are vital in veterinary practices and the wider animal welfare professions.

The Animal Welfare Assessment Grid (AWAG) is a tool that monitors the welfare of animals and has been validated across a range of species (38–42). The AWAG assesses physical health, psychological wellbeing, environmental comfort, and veterinary and managemental procedural events. The tool also monitors the cumulative lifetime experience of the animal by assessing the animal throughout its lifetime which can be done in real time or through the use of retrospective and prospective assessment This is important from a welfare perspective as the cumulative impact of positive and negative experiences determines an animal's quality of life (43) and these can shape cognitive bias and long-term emotional state. The duration of positive and negative experiences and the intervals between events must also be taken into account as these can also have a lasting impact on welfare (44). The AWAG has been tested using both retrospective and in-life data and has been found to give a clear indication of animals' welfare during their lifetime (26).

When an animal is scored, the four parameters are visualized with grid scores plotted on the four axes across on a radar chart. By having this, it provides a numeric and visual representation of the animal's welfare state, and if significant changes in welfare are seen, the tool can show which factors have contributed to these changes. Therefore, intervention can be specifically focused to improve the animal's wellbeing.

Each parameter (physical, psychological, environmental, and procedural) is subdivided into several factors that contribute to the overall score. For example, the physical score would encompass the patient's general condition, clinical assessment, pain control, inappetence, and activity level.

Within each parameter the various factors are scored between one and ten. Each factor score is defined using descriptors for each number to reduce scoring bias. A score of one indicates the best possible state (lowest possible impact on welfare), whilst a score of 10 would be the worst possible state (highest possible impact on welfare), for each respective factor. For each parameter, mean factor scores are then calculated and this allows the clinician to ascertain what parameters are impacting quality of life at that time point.

**Figure 1:**
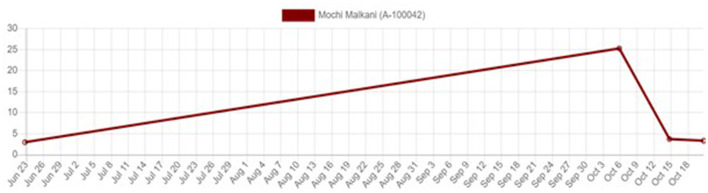
Cumulative Welfare Assessment Score over time.

**Figure 2:**
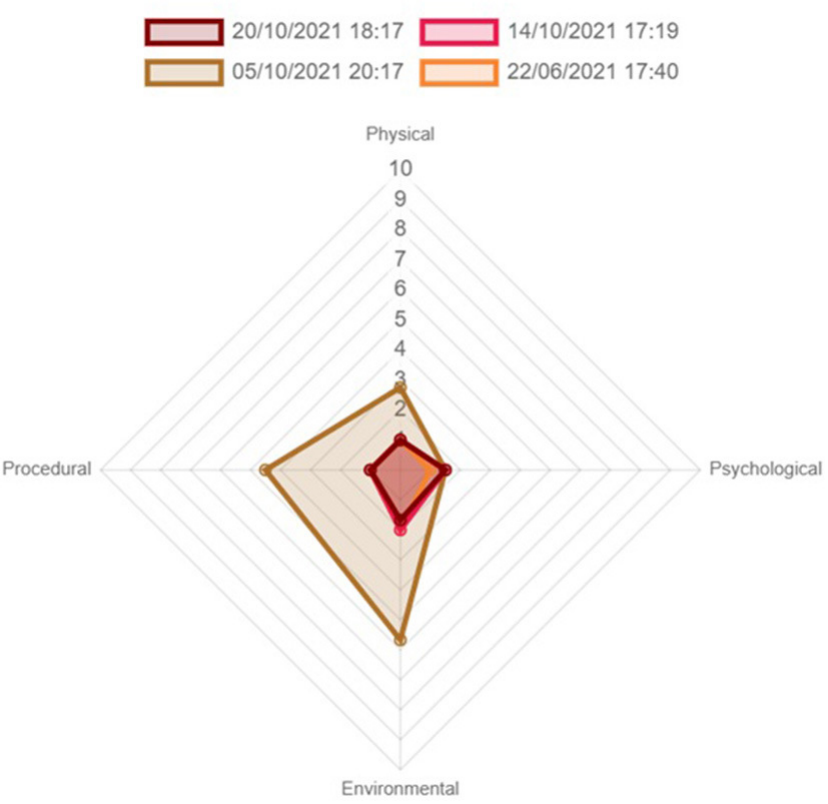
Cumulative Welfare Score plotted on the axis of the four parameters.

In addition to the ability to quantify quality of life at a given time point, the tool provides a visual representation of the animal's cumulative welfare state (Figure 1). The radar chart displays the four parameters. Each parameter score is calculated from the factors scores, and the resultant scores are marked on the x and y axis. These points are joined together to create a polygon and the total area covered is calculated to derive the cumulative welfare assessment score (CWAS) at that particular point in time (Figure 2).

If significant changes in welfare are seen, the tool can show which factors have contributed to these changes and intervention can be undertaken to improve the animal's wellbeing. This is particularly important in hospitalized patients as it allows veterinary staff to assess the welfare impact of clinical interventions, environment, physical and emotional health to determine what factors are influencing welfare. Veterinary staff can then enhance the factors that contribute to positive welfare and reduce or change elements that are negatively impacting welfare. Additionally, prospective assessments can be undertaken to examine how different treatments will impact the dog's welfare, allowing for improved decision-making.

The aims of this study were to develop an online, easy to use platform for the AWAG and to adapt the AWAG for dogs and conduct initial validity and reliability testing; thus creating an objective, evidence-based welfare assessment tool for anyone working with dogs to use.

## Methods

### Development of the online AWAG platform

The AWAG software was designed in partnership with Reuben Digital Ltd. ™, Wiltshire and the development of the site began in June 2020. The platform was designed to be functional on a range of devices such as desktops, laptops, tablets and mobiles. Data that were needed to be captured for functionality and analysis were established. The site was designed to easily capture demographic data such as dog name, age, breed, neutered status, and diagnosis using dropdown and free-text functions. The tool was also developed in a way that the assessor can enter events at a certain date and time such as a medical procedure or change in environment as these may provide insight into why a dog's welfare state has changed.

### Factor descriptors

#### Design of the factors and their descriptors

In order to design and adapt the AWAG for dogs, the individual factors to be scored across each parameter (physical, psychological, environmental, and procedural) and the written descriptors needed to be determined. From reviewing the literature, several factors that can contribute to each welfare parameter were identified that influence a dog's quality of life or that may result in cumulative suffering. When deciding on which factors to score, it was important to assess if these would be feasible to score as well as give information about a dog's wellbeing.

The primary physical factors identified in the literature were mobility, conformation, body condition (overweight or underweight), food and water consumption, health state and comorbidities. The physical assessment that the clinician undertakes will encompass the presence of disease, illness, injury, and pain and its likely impact on welfare, which can be further affected by a dog's conformation. A dog's body condition is an objective measure of their physical health status and this is primarily affected by their intake of food. Reduced food intake is an important clinical sign that can result from a myriad of chronic diseases (45). Ill dogs often show reduced food intake or anorexia. Moreover, dogs that are anxious or fearful are often anorexic (46, 47). Therefore, assessing an individual's food intake may give insight into the individual's quality of life.

Psychological factors that can affect general welfare in a negative way include past experiences, fears, and anxieties; specifically, negative experiences in the veterinary practice, intolerance to being handled, separation distress, fear of people, fear of dogs, and fear of noises. Methods of coping such as reactivity and aggression toward stressors can be used to indicate poorer states of welfare.

There are many aspects of a dog's environment that the literature suggests are important to dogs and include the facilitation of a social environment that involves positive interaction with people and other dogs, the opportunity to play, and the ability to use choice and control their environment. As dogs' social needs are so individual, the factor scale describes dogs that have positive social interactions that match their emotional needs. For example, one dog may need frequent engagement with people and other dogs and/or other species throughout the day to meet to maintain emotional wellbeing, whereas another dog may prefer the company of one person and time alone to meet their welfare needs. Dogs that don't encounter other humans or dogs due to fear or dogs that have attachment issues (48) are also included alongside a lack of social environment, as both of these situations will result in poorer welfare scores. As the opportunity to play is intertwined with how enriched the dog's environment is, these were also combined into one scale.

Elements of procedures and management that can affect a dog is the likelihood of pain, handling, length of hospitalization or time in a restricted environment, and the impact on routine. All veterinary procedures will have some impact on welfare which may be short-term and minimally affect the dog, or longer-term involving sedation or anesthesia and affecting the dog's daily routine and ability to carry out normal functions and behaviors.

Peer-reviewed literature and data that report the presentation and severity of the aforementioned welfare factors were used to shape each scale. Descriptors were written and refined for each score. This enabled the respondent to answer on an objective scale of one (least severe) to ten (most severe), which was undertaken to reduce respondent scoring bias by defining each score comprehensively (Current factors—Table 3).

**Table 3:** Factors scores and descriptors.

**Physical**			
**Mobility**	**Body condition**	**Clinical assessment**	**Eating and drinking**
1. the dog has very good mobility with no lameness or stiffness and is normally active or has normal energy	1. ribs easily palpable without pressure, with minimal fat covering, waist easily noted and evident abdominal tuck	1. clinically healthy, no injury or sign of disease	1. eating and drinking as normal
2. very good mobility with occasional mild stiffness and is normally active	2. ribs fairly easy to palpate without pressure with thin fat covering and evident abdominal tuck from above	2. mild transient subclinical symptoms or injury but has no evident behavior change or impact on welfare	2. food and/or water consumption is minimally reduced
3. good mobility with short bouts of stiffness	3. slight fat covering, slight pressure needed to palpate ribs, waist observable from above	3. mild transient clinical symptoms or injury with mild transient behavior change and impact on welfare	3. mild to moderate reduced food/water (>20%)
4. good mobility with generalized stiffness	4. slight covering of fat, slight waist observable from above, can palpate ribs with pressure needed	4. mild clinical symptoms or injury with mild behavior change and impact on welfare	4. moderately reduced food/water (>30%)
5. moderate mobility, stiffness but frequently active	5. moderate covering of fat, waist discerned from above but not prominent, can palpate ribs with pressure	5. moderate transient clinical symptoms or injury with some behavior change and impact on welfare	5. moderately reduced food/water (>50%)
6. moderate mobility, stiffness and less active	6. excess covering of fat, no discernible waistline and difficulty palpating ribs	6. moderate clinical symptoms or injury with moderate behavior change and impact on welfare	6. severely reduced food/water (>80%)
7. poor mobility, stiffness and less active	7. (overweight) heavy fat present and slight abdominal distension, difficult to palpate ribs or (underweight) ribs and shoulder visible with little fat	7. moderate/severe disease or injury with moderate behavior change and impact on welfare	7. anorexic, has minimal loss of skin turgor
8. very poor mobility, stiffness and minimally active	8. (overweight) heavy fat present with abdominal distension, cannot palpate ribs or (underweight) ribs, lumbar vertebrae and pelvic bones somewhat visible with little detectable fat	8. moderate/severe disease or injury with severe behavior change and impact on welfare	8. anorexic, has moderate loss of skin turgor, somewhat dry mucous membranes
9. very poor mobility, stiffness and not at all active	9. (overweight) very heavy fat present with obvious abdominal distension, cannot palpate ribs or (underweight) ribs, lumbar vertebrae and pelvic bones easily visible with very little fat	9. severe disease and clinical symptoms or injury with severe of behavior change and impact on welfare	9. anorexic, has considerable loss of skin turgor, dry mucous membranes OR severe hunger/thirst
10. Non-ambulatory and cannot move without assistance or support	10. Massive fat deposits over neck thorax, spine, limbs and base of tail with obvious abdominal distention, cannot palpate ribs or ribs, lumbar vertebrae, pelvic bones and all bony prominences evident from a distance. No discernible body fat and obvious loss of muscle mass	10. Extreme disease and clinical symptoms or injury with extreme behavior change and impact on welfare	10. Anorexic, has major loss of skin turgor, extremely dry mucous membranes OR severe and constant hunger/thirst
**Psychological**		
**Aggression toward caregiver**	**Aggression toward unfamiliar people**	**Fears and anxieties frequency**	**Reaction to stressors**
1. none	1. none	1. rarely encounters stressors	1. displays minimal signs of fear and anxiety when encounters potential stressors
2. occasionally growls, is predictable and trigger avoided	2. occasionally growls, is predictable and trigger avoided	2. encounters stressors a couple of times a year	2. shows signs of fear to stressors and returns to normal <30 s
3. occasionally growls, is predictable but trigger not always avoided	3. occasionally growls, is predictable but trigger not always avoided	3. encounters stressors multiple times a year	3. shows signs of fear to stressors and returns to normal in minutes
4. occasionally growls, is predictable but trigger rarely avoided	4. occasionally growls, is predictable but trigger rarely avoided	4. encounters stressors monthly	4. shows signs of fear to stressors and some minor and returns to normal after 10 minutes
5. occasionally snaps or bites, is predictable and trigger avoided	5. occasionally snaps or bites, is predictable and trigger avoided	5. encounters stressors weekly	5. shows signs of fear to stressors and returns to normal after 30 min
6. occasionally snaps or bites, is predictable but trigger not always avoided	6. occasionally snaps or bites, is predictable but trigger not always avoided	6. encounters stressors several times weekly	6. shows signs of fear to stressors and takes up to an hour to return to normal
7. occasionally snaps or bites, is predictable but trigger rarely avoided	7. occasionally snaps or bites, is predictable but trigger rarely avoided	7. encounters stressors daily	7. shows signs of fear to stressors and takes several hours to return to normal
8. bites, is somewhat predictable and trigger largely avoided	8. bites, is somewhat predictable and trigger largely avoided	8. encounters stressors over 50% of the day	8. shows signs of fear to stressors and takes most of the day to return to normal
9. bites, is somewhat predictable and trigger not avoided	9. bites, is somewhat predictable and trigger not avoided	9. encounters stressors over 75% of the day	9. shows signs of fear to stressors and takes several days to return to normal
10. severe bites that are unpredictable	10. severe bites that are unpredictable	10. encounters constant stressors	10. shows signs of fear to stressors and is always anxious
**Environment**		
**Choice, control, and predictability**	**Enrichment**	**Social**
1. has good control over their environment and can make a range of choices, has highly predictable environment	1. engaged in multiple forms of enrichment for over 2 h daily	1. has high-quality social interactions daily
2. has good control over their environment and can make a range of choices, mostly has predictable environment	2. engaged in multiple forms of enrichment for 1–2 h daily	2. has high-quality social interactions most days
3. has some control over environment, can make some choices, has mostly predictable environment	3. engaged in multiple forms of enrichment for up to 1 h daily	3. has good-quality social interactions daily
4. has some control over environment, can make some choices, has some predictability	4. engaged in enrichment for up to 30 mins daily	4. has good-quality interactions most days
5. has little control over environment, can make some choices, has little predictability	5. engaged with enrichment for <15 mins daily	5. has good-quality interactions weekly
6. spends several hours in an unpredictable environment, can make some choices	6. somewhat engaged with enrichment several times weekly	6. has moderate-quality interactions weekly
7. spends time in an unpredictable environment, can make few choices	7. somewhat engaged with enrichment weekly	7. the dog is socially isolated most days and has moderate-quality interactions in between
8. spends time in an unpredictable environment, can make few choices	8. poorly engaged with enrichment monthly	8. the dog is socially isolated most days and has poor social interactions in between
9. spends time in an unpredictable environment, can make very few choices	9. rarely engages with any forms of enrichment	9. the dog is socially isolated for 50% of each day and has poor social interactions the rest of the time
10. spends almost all of their time in highly unpredictable environment, cannot make any choices	10. has no enrichment or does not engage with enrichment	10. the dog is constantly socially isolated
**Procedural**			
**Behavior during assessment**	**Change in daily routine**	**Handling during assessment**	**Procedure pain**
1. is calm and actively seeks interaction from assessor/s	1. Procedure/disruption to day <15 min	1. displays minimal signs of stress when handled, is calm and tolerates being handled well	1. no procedure required
2. is mostly relaxed and shows mild signs of stress to few triggering events	2. Procedure/disruption to day <30 min	2. minimal movement when handled, sometimes licks lips, yawns or shows other appeasement behavior	2. minor procedure with no expected pain
3. is somewhat relaxed and shows mild signs of stress to some triggering events	3. Procedure/disruption to day 30 mins−1 h	3. minimal movement when handled, licks lips, yawns, or shows appeasement behavior frequently	3. minor procedure longer duration with no expected pain
4. is not relaxed and shows moderate signs of stress to few triggering events	4. Procedure/disruption to day 1–2 h	4. some slow movement when handled, turns head away from handler, slow panting, displays more than two signs of stress such as ears back and tail down	4. minor procedure with short mild pain
5. shows moderate signs of stress to some triggering events	5. Procedure/disruption to day 3–4 h	5. moderate movement when handled, fast panting, displays more than two signs of stress such as ears back, tail tucked and furrowed brow	5. moderate procedure with short duration of transient pain
6. shows moderate signs of stress to all triggering events	6. Procedure/disruption to day >4 h	6. some attempt to escape, fast movements, tense body and tense closed mouth	6. moderate procedure, longer in duration with transient pain
7. shows major signs of stress to few triggering events	7. Procedure/disruption to day > 6 h	7. moderate attempts to escape, fast movements or frozen and staring, tense and trembling	7. moderate/severe procedure, with pain lasting >12 h
8. shows major signs of stress to some triggering events	8. Procedure/disruption to day >8 h	8. strong attempts to escape when handled or frozen, lifts lips and shows teeth	8. severe procedure with pain lasting >24 h
9. shows major signs of stress to all triggering events	9. Procedure/disruption to day >12 h	9. will violently attempt to escape when handled or frozen, growls and barks	9. severe procedure with pain or complications lasting > 48 h
10. cannot cope being in the environment, is extremely shut-down or aggressive and shows major signs of stress	10. Procedure/disruption to day >24 h	10. cannot be handled, growls and attempts to bite when approached	10. extensive procedure resulting in severe long-term pain or complications

Definitions and examples for terms that may be ambiguous such as “signs of stress”, “enrichment”, and “predictability” were written and implemented into on the AWAG site for user clarification (Table 4).

**Table 4:** Terms used in the AWAG factor descriptors and their definitions.

**Term**	**Definition and examples**
Choices and control	The ability to choose where to sleep, rest, and visualize their environment from different vantage points. Decide the activities and interactions that they engage in.
Enrichment	Addition to the environment that enhances the dog's mental state—exercise, sensory toys, feeding devices etc.
Good-quality social interactions	Indirect engagement—walking/resting
High-quality social interactions	Direct engagement—play/agility/training /
Predictability	Can accurately expect the consequences of actions and has a regular routine
Shut-down	Reduced responsiveness/disconnected/depressed but not relaxed
Signs of stress	Behavioral indicators that the dog is attempting to cope with a stressor and feeling in a state of unease

#### Recruitment

A multi-pronged recruitment strategy was employed to access veterinary networks as widely as possible across the UK. Recruitment posters were sent to the University of Surrey partner practices and were placed in the Veterinary Times journal. Information about the project was also distributed to professional networks and recruitment posters were shared on social networking sites including Facebook, Twitter, and LinkedIn; followers of these pages were able to share the link if they wished to.

#### Consent

Prior to being provided with a login to the AWAG site, users were required to submit a signed consent form outlining the research. Clinicians also required owners to sign consent forms. If dogs were in a shelter or other environment where they were not “owned”, the main contact for the organization was required to sign a consent form for all the dogs in their care. Clinicians and owners were also provided with information sheets that provided further details about the research project and data storage and security.

#### Pilot studies

Pilot studies were undertaken to gain feedback on the functionality and factor scores of the AWAG tool. Twelve clinicians including veterinarians, veterinary behaviorists, and European specialists in behavior piloted the tool and provided both quantitative and qualitative feedback. Factor scores and the functionality of the site were refined on the basis of feedback from these clinicians.

#### Validation studies

The clinical usefulness of an instrument depends on its ease of use and on its validity. It is important to note that validity is not a dichotomous variable, but a continuous one. The more evidence that can be provided for an instrument, the more “valid” it becomes (49). Thus, following the pilot studies and refinement of the tool, further studies were carried out to ensure the tool was valid and reliable.

These studies tested the tool to ensure experts agreed the factors scored are suitable to assess dog welfare (content validity), that the tool can differentiate between dogs in good and poorer states of welfare (construct validity), and that it produces the same score under non-changing conditions (test re-test reliability). This is important in a welfare assessment tool such as the AWAG as it is necessary to establish that it can represent and quantify an animal's welfare state accurately and that the scores change appropriately where welfare improves or worsens. If the tool is not well validated, it may give a misrepresentation of a dog's wellbeing, resulting in suffering if necessary interventions are missed. Testing reliability is also vital to ensure that the AWAG gives consistent scores under non-changing conditions and that there is little to no variability in the scores between different users, otherwise changes in welfare may not be detected, or conversely, it may show changes in welfare where there are none.

#### Suitability of factor scores (content validity)

A standard method for assessing content validity involves judgments by subject matter experts (SMEs) with expertise in the content of the test. The Content Validity Index (CVI), a proportion agreement procedure, allows two or more raters to independently review and evaluate the relevance of a sample of items to the domain of content represented in an instrument. The recommended number of experts to review an instrument varies from two to 20 individuals and least five people are suggested to review the instrument to have sufficient control over chance agreement (50).

**Figure 3:**
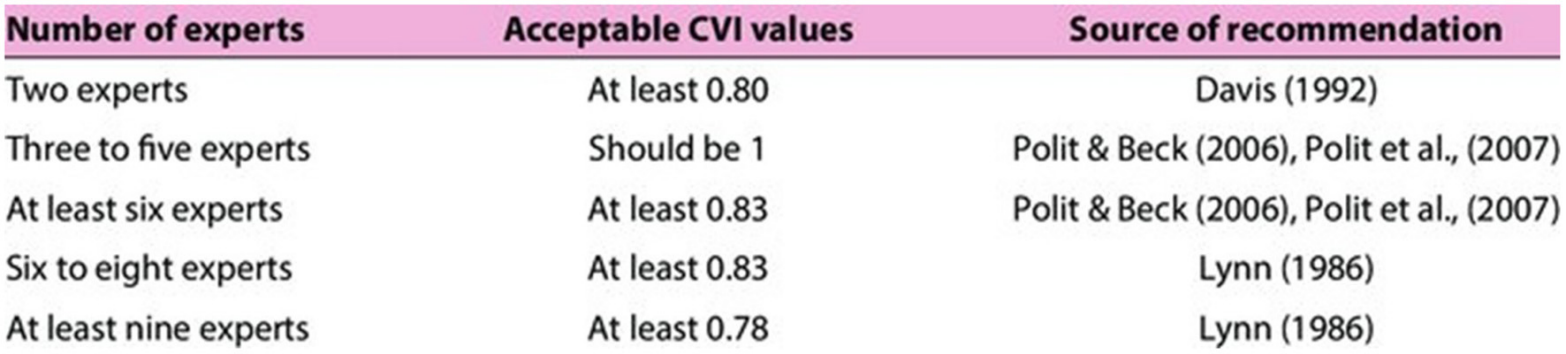
Acceptable CVI values.

In order to ensure the factor scores and descriptors were suitable to assess dog welfare ‘Subject Matter Experts' (SMEs) (*n* = 7) reviewed each factor and rated whether they felt the factor was 1 = not relevant, 2=somewhat relevant, 3= quite relevant, or 4 = very relevant to the assessment of dog welfare. Ratings of 1 and 2 are considered “content invalid,” whereas ratings of 3 and 4 are considered to be “content valid” (51, 52). SMEs were deemed to be experts in dog welfare if they met one of the criteria as described in Table 5. Yusoff provide this table (Figure 3) that outlines acceptable content validity index (CVI) values (53).

**Table 5:** Subject matter expert in dog welfare criteria.

**Criteria number**	**Criteria**
1	Veterinary surgeons that were Diplomates in Animal Welfare Science, Ethics and Law (AWSEL), Behavioral Medicine (BM) or both. This means they have undergone extensive training programme over several years within the fields of animal welfare and behavioral medicine before passing a board examination.
2	Veterinary surgeons or veterinary nurses with extensive experience working in dog welfare that have received awards for services to animal welfare such as an MBE or OBE.
3	Veterinary surgeons who are advanced practitioners or who have extensive experience working in dog welfare and have been granted Fellowship to the Royal College of Veterinary Surgeons for recognition of outstanding contributions to the veterinary profession.
4	Animal welfare professionals with extensive experience working in dog welfare with advanced qualifications (PhD) in dog welfare.

#### Ensuring the AWAG measures different welfare states (construct validity)

Construct validation examines the extent to which a measure assesses the construct that it is intended or supposed to measure (54). This is fundamental in a welfare assessment tool as it is essential that the instrument can report welfare accurately and that the scores increase as welfare states worsen. This trial was conducted by veterinary and animal welfare professionals using the AWAG to score a cohort of dogs that they have evaluated through consultation and examination to be emotionally and clinically healthy to obtain baseline scores for healthy dogs. Assessors also scored healthy dogs undergoing neutering as this is a procedure that is well-known to impact welfare negatively in the short-term as a result of starvation, hospitalization, pain, restricted choice and exercise. Additionally, users scored a cohort of dogs with chronic disease as it is likely that dogs with chronic conditions will score poorer due to the impact these conditions can have on quality of life.

#### Testing scores under non-changing conditions (test re-test reliability)

Reliability estimates using a test–retest approach measure the degree to which the same testing instrument produces similar results when administered to the same individual in as similar a manner as possible over a period of time. Test–retest reliability is a popular form of reliability estimation for the development and validation of test instruments and is based on correlation (55). Polit (56) state that retest reliability coefficients that approach or exceed 0.80 in their field tests are recommended and (57).

In order to assess test re-test reliability, individual clinicians conducted multiple assessments of 19 emotionally and medically healthy dogs (some in the cohort of healthy dogs used in construct validity testing) in stable and non-changing environments over a two-week period. They performed a minimum of two and a maximum of five tests using the AWAG.

#### Assessing scores of users on the same patient (inter-rater reliability)

To evaluate the reliability of consistency of scores between users when assessing the same patient, dogs were assessed by two users using the AWAG at the same time in the veterinary clinic during the consultation or when they were hospitalized. In two assessments one user had missed scoring a factor that the other user had scored, so this data was discarded as it would have been unreliable.

### Data analysis

#### Power analysis

Power analysis was undertaken to calculate an appropriate sample size to assess construct validity in the AWAG. An effect size was calculated in R Studio using the “effect size” package with previous data on healthy and chronically ill dogs. A sample size for the number of dogs was then calculated using G^*^Power 3.1.

### Statistical analysis

#### Test re-test and inter-rater reliability

Highest and lowest scores over multiple assessments were used to assess the variation in scores. Pearson's correlation was used in R Studio to test if the scores were correlated. Analysis of Variance test (ANOVA) was also used to assess the variation between the repeated test scores for each individual dog. This was undertaken in R Studio using the “datarium” package.

#### Content validity

To calculate item-level content validity index (I-CVI), the relevance rating for each factor was coded to either zero as not relevant, or one as relevant. The number of ones was totaled and divided by the number of experts. The S-CVI/Ave was calculated using the total of the average of the I-CVI ratings divided by the number of factors.

#### Construct validity

Shapiro-Wilk tests were conducted to test the normality of the data from healthy dogs, sick dogs, and dogs undergoing neutering. Mann Whitney tests were used to compare scores of healthy dogs with sick dogs and healthy dogs with dogs having neutering procedures.

## Results

### Pilot studies

**Figure 4:**
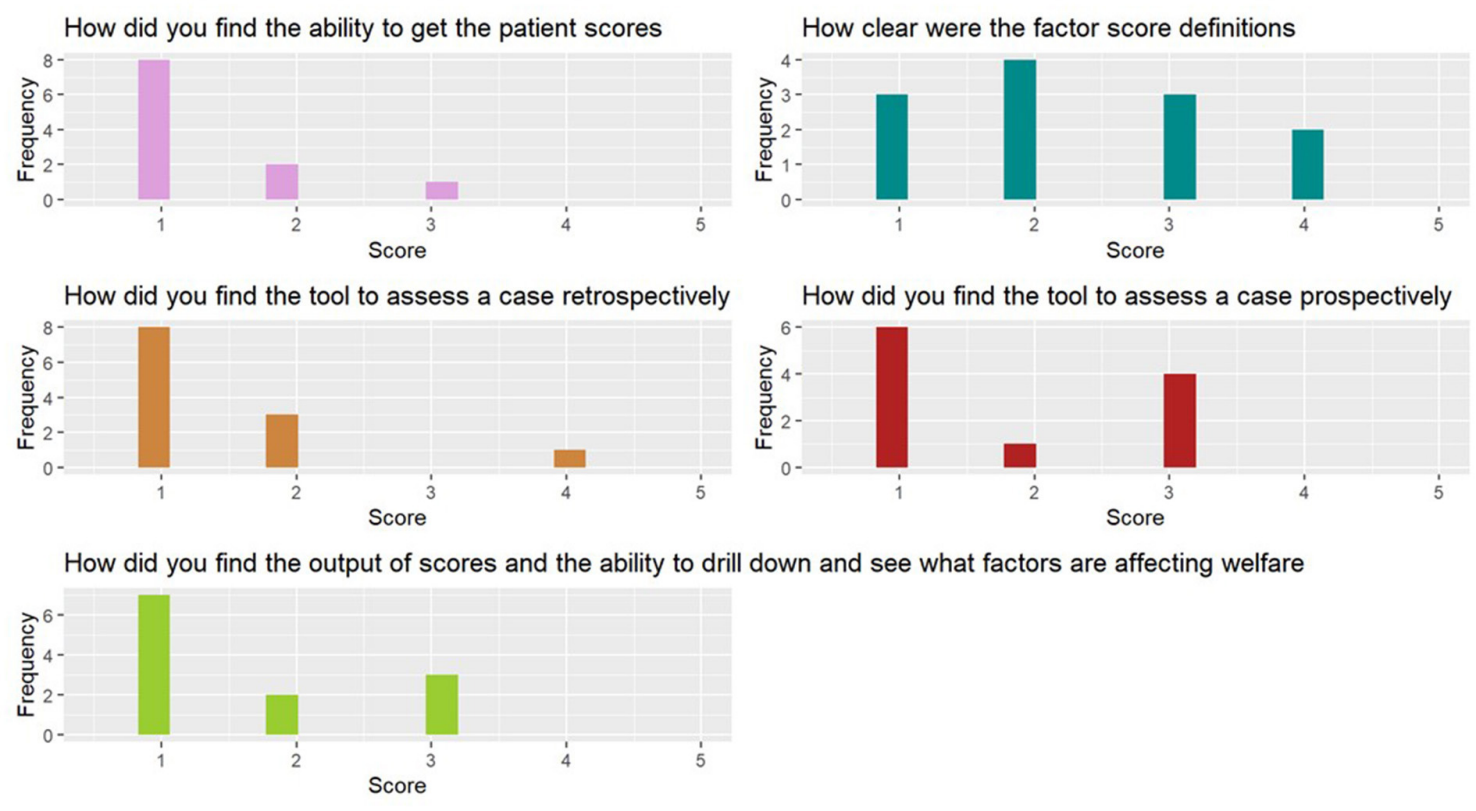
Likert-scale question scores (one = very easy, five = very difficult).

Results of the Likert-scale questions that obtained data on the factor scores and functionality can be seen in Figure 4.

Feedback from clinicians from the pilot study reported that users thought the tool was important and that the reporting elements were useful, but the tool could be more intuitive with clearer buttons and section headings.

Users of the AWAG felt that the factors were important for dog welfare. One user felt a factor that was missing was side effects of the treatment. However, more people believed some factors were unnecessary. Therefore, the factors were also refined down from 22 to 16. Factors that were removed include comorbidities as these would be picked up by the clinical assessment score, rendering comorbidities unnecessary. Sedation/anesthesia scores were also removed as the sedation or anesthetic element does not impact alone; the associated impacts on routine, eating and drinking, and pain are the primary welfare considerations, and these are being scored as separate factors. Travel to the veterinary practice, separation distress, and abnormal behaviors were also removed as these will be captured within other factors (Table 6).

**Table 6:** Factors removed during validation and refinement of the AWAG for dogs.

**Parameter**	**Factor**
Physical	Comorbidities
Psychological	Abnormal behaviors
	Separation distress
Procedural	Separation distress in veterinary practice/management environment
	Sedation/anesthesia
	Travel

Regarding the factor descriptors, the general feedback was that they were too detailed, and some were not mutually exclusive. The descriptors were amended in order to address this and were written in a more basic and simpler format.

### Power analysis for construct validity

The power analysis *t-*tests report that a total sample size of 48 dogs is needed to differentiate between healthy and sick dogs for use in validation testing.

### Content validation

All seven of the SMEs were in agreement that each factor was relevant to assessing dog welfare besides “aggression toward unfamiliar people” where six out of the seven SMEs believed this was relevant to dog wellbeing. The I-CVI (the proportion of content experts giving item a relevance rating of 3 or 4) = 0.99 (acceptable score 0.83) and S-CVI/Ave (sum of proportion relevance rating)/(number of expert) = 0.94 (Table 7), demonstrating that SMEs agree that the factors scored are representative of a dog's welfare and the AWAG is considered a valid tool to assess wellbeing.

**Table 7:** Relevance ratings and scores calculated by SMEs (U = user, I-CVI = proportion of content experts giving item a relevance rating of 3 or 4, UA = users in agreement, S-CVI/Ave = (sum of proportion relevance rating)/(number of experts).

**Factor**	**U1**	**U2**	**U3**	**U4**	**U5**	**U6**	**U7**	**Experts in agreement**		**I-CVI**	**UA**
Mobility/activity	1	1	1	1	1	1	1	7		1	1
Clinical assessment	1	1	1	1	1	1	1	7		1	1
Body condition	1	1	1	1	1	1	1	7		1	1
Eating and drinking	1	1	1	1	1	1	1	7		1	1
Aggression toward caregiver	1	1	1	1	1	1	1	7		1	1
Aggression toward unfamiliar people	1	1	1	1	0	1	1	6		0.857143	0
Fears and anxiety frequency	1	1	1	1	1	1	1	7		1	1
Reaction to stressors	1	1	1	1	1	1	1	7		1	1
Separation distress	1	1	1	1	1	1	1	7		1	1
Social	1	1	1	1	1	1	1	7		1	1
Enrichment	1	1	1	1	1	1	1	7		1	1
Choice, control, and predictability	1	1	1	1	1	1	1	7		1	1
Behavior during assessment	1	1	1	1	1	1	1	7		1	1
Change in daily routine	1	1	1	1	1	1	1	7		1	1
Handling	1	1	1	1	1	1	1	7		1	1
Procedure pain	1	1	1	1	1	1	1	7		1	1
									**I-CVI=**	**0.991071**	15
									**S-CVI/AVE=**	**0.9375**	

### Construct validation

#### “Healthy” vs. “sick” dogs

**Figure 5:**
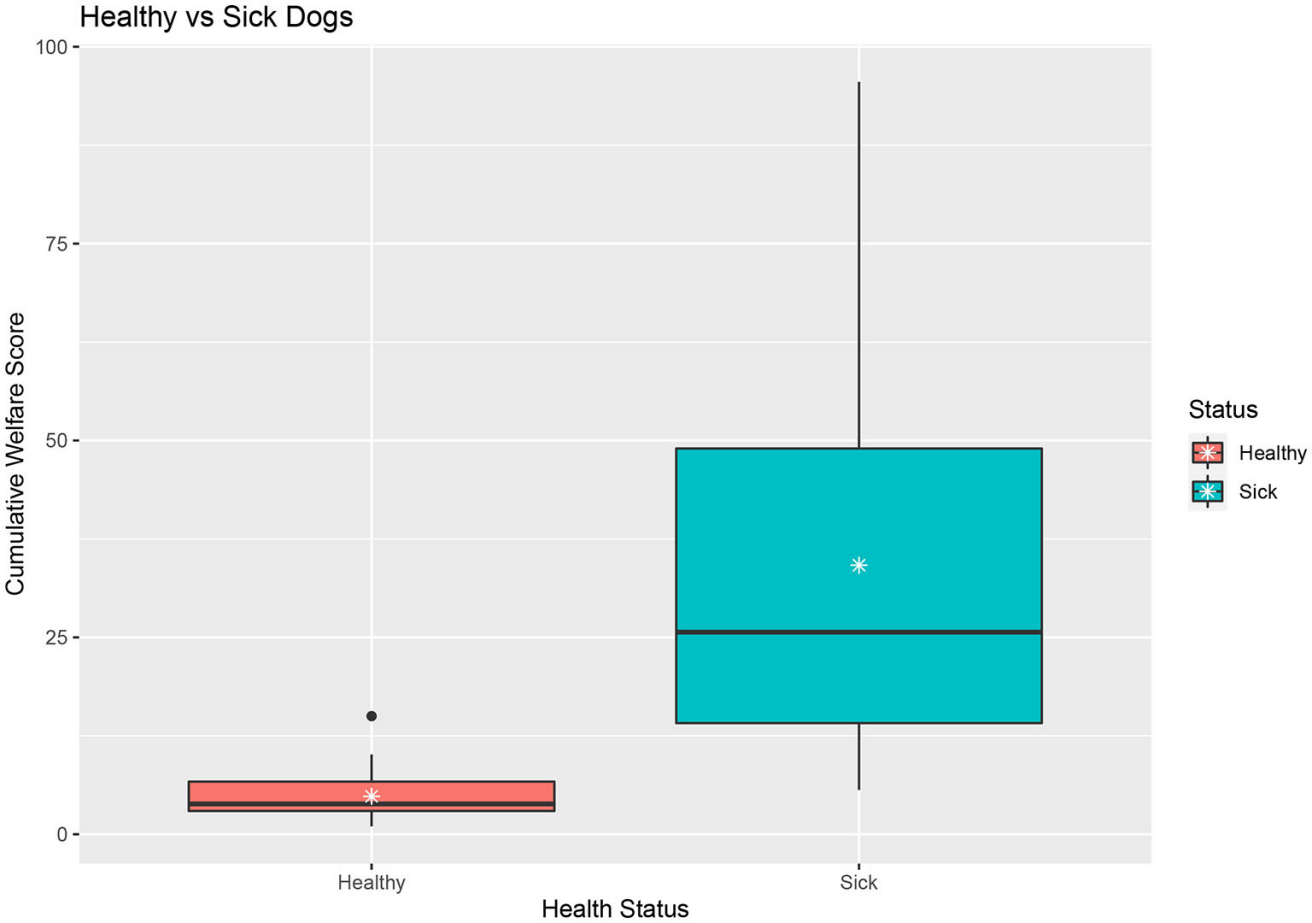
Boxplot of healthy dogs compared to sick (chronic condition) dogs.

Mann Whitney tests assessed the difference of Cumulative Welfare Score by health status (mean in group Healthy = 4.78, mean in group Sick = 34.16) suggests that there is a statistically significant difference between the scores of healthy (*n* = 41) and sick dogs (*n* = 47) (*W* = 46, *p*-363 value = < 0.001) (Figure 5).

#### “Healthy” vs. “routine procedure” dogs

**Figure 6:**
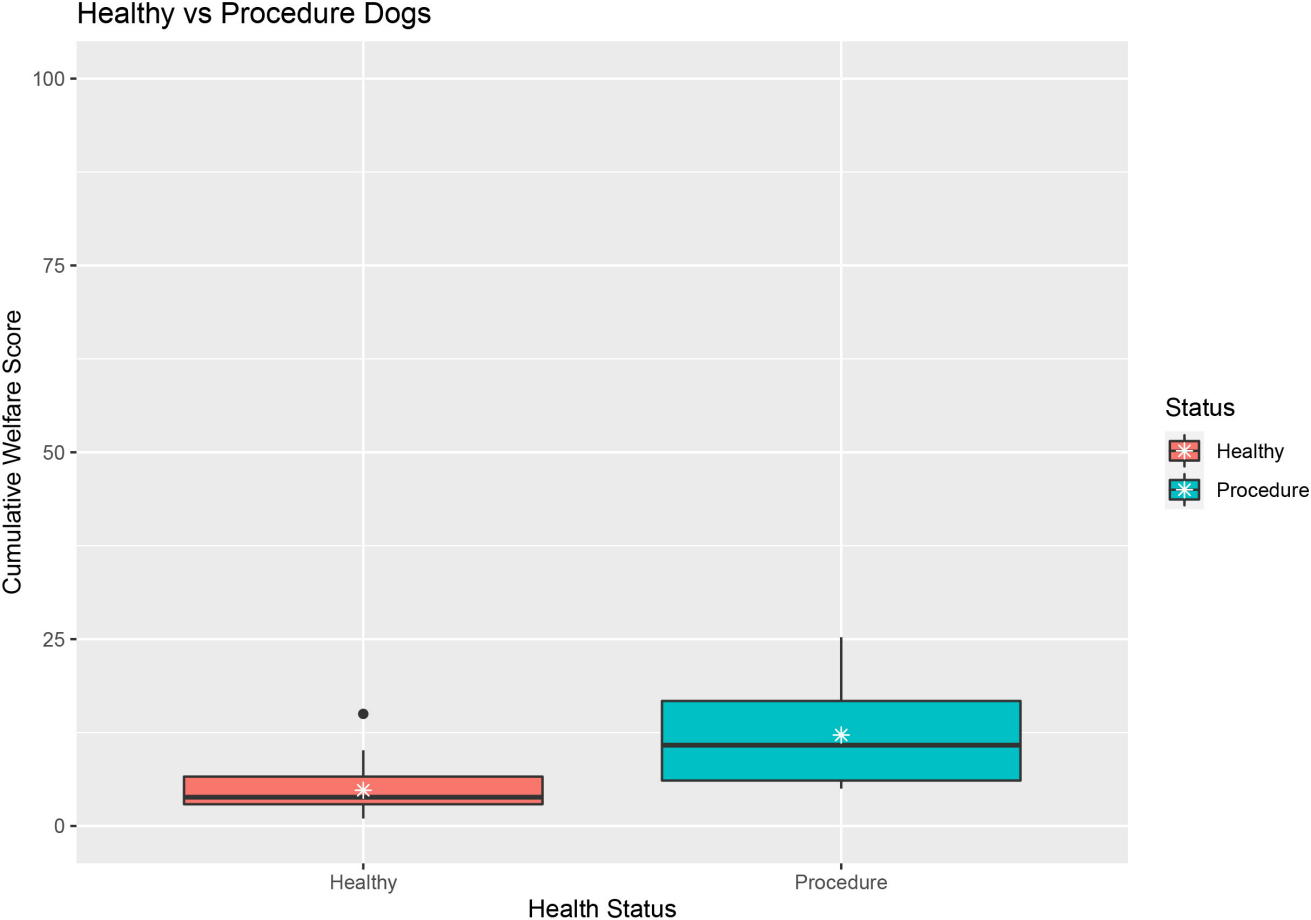
Boxplot of healthy dogs compared to dogs hospitalized for neutering.

Mann Whitney tests assessed the difference of Cumulative Welfare Score by health status (mean in group Healthy = 4.78, mean in group procedure = 12.17) suggests that there is a statistically significant difference between the scores of healthy (*n* = 41) and dogs undergoing routine procedures (*n* = 8) (*w* = 45, *p*-value = < 0.001) (Figure 6).

### Reliability

#### Test re-test reliability

**Figure 7:**
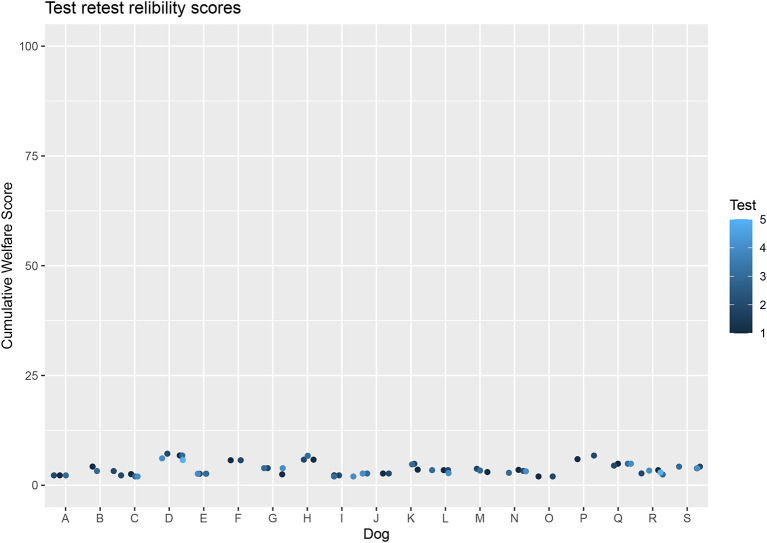
Cumulative Welfare Assessment Score of dogs in test re-test reliability study.

The highest and lowest scores for individual dogs were shown to be strongly correlated (*p* = <0.001, *r* = 0.89) and repeated measures ANOVA report no significant difference between tests for each dog (*F* = 0.55, *p* = 0.71). These demonstrate that there is little variation in the scores of dogs repeatedly tested in non-changing conditions (Figure 7).

#### Inter-rater reliability

**Figure 8:**
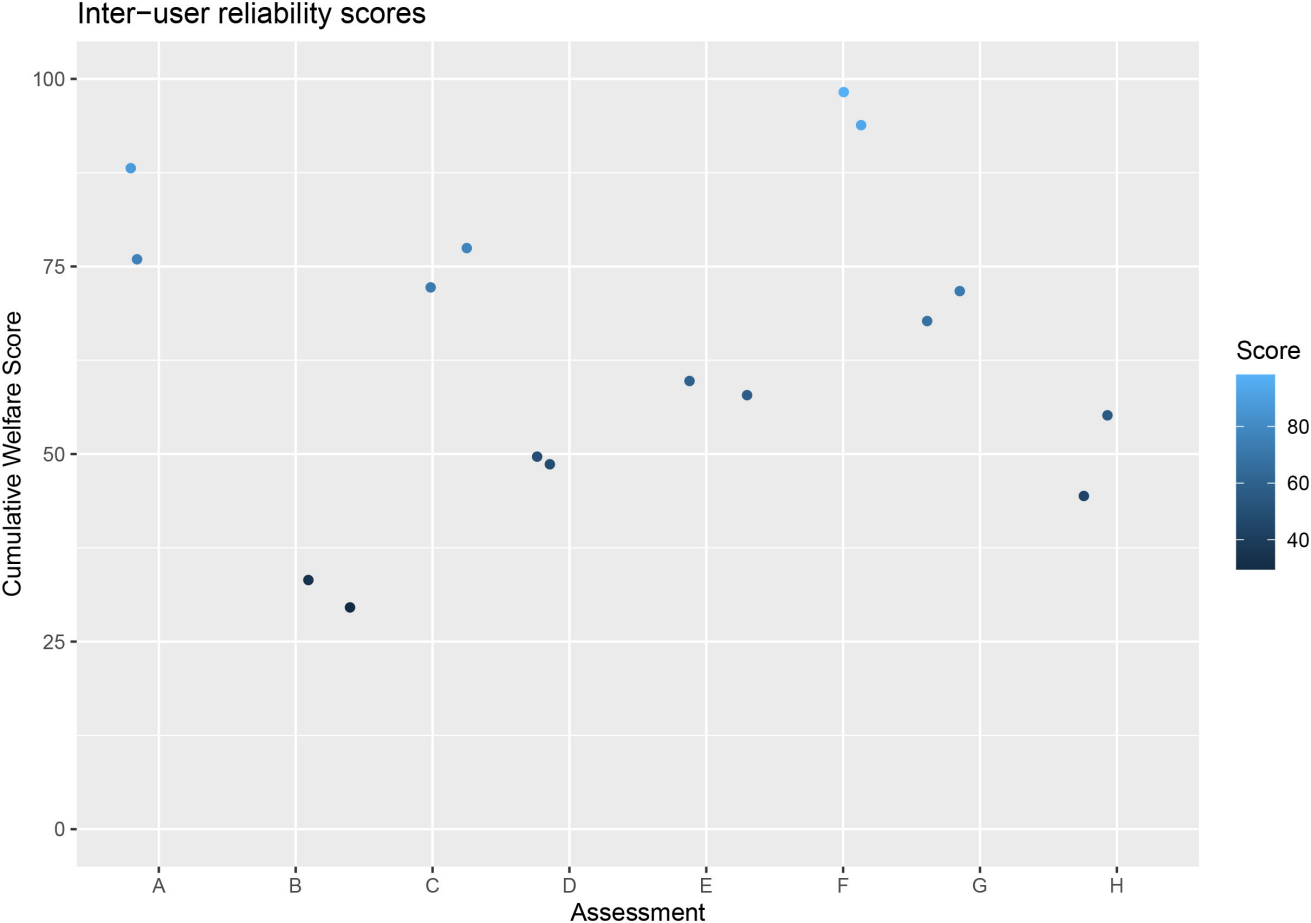
Cumulative Welfare Assessment Scores of dogs in inter-rater reliability study.

The two scores for each dog were shown to be highly correlated (*p* = <0.001, *r* = 0.97) and repeated measures ANOVA shows no statistical difference between scores (*F* = 0.39, *p* = 0.55) (Figure 8).

## Discussion

This project involved the development of the AWAG for dogs into a novel, evidence-based, online platform for veterinary and animal welfare professionals to holistically assess canine welfare. Additionally, this study aimed to provide measures of validation and reliability to assess whether the AWAG could accurately measure canine welfare and to ensure the tool was reliable and consistent. The results of this research suggest that the AWAG for dogs is a valid, reliable, and easy-to-use tool for clinicians to score the welfare of the dogs in their care.

The result of the pilot trials provided valuable data on improving both the functionality of the tool and the factor scores. As a large proportion of clinicians believed that many of the initial factors on the system were unnecessary, this helped reduce the number of factors scored. It is important that tools for dog welfare professionals are quick and easy to use due to time constraints in animal welfare settings; therefore, having fewer factors to score made the tool more practical to use in practice. However, it was important to find balance between making the AWAG quick and easy to use whilst being comprehensive enough to accurately assess welfare. Conducting content validation of the tool demonstrated that experts in canine welfare scored each factor to be either quite relevant or very relevant to assess welfare in dogs under the respective parameters.

Pilot trials also revealed that the factor score descriptors could be clearer, with 17% scoring the factor score definitions as difficult. Quantitative feedback reported that some factor descriptors were not always mutually exclusive. The factor descriptors were refined to be simpler, clearer and mutually exclusive.

Following feedback from veterinarians on the factors from pilot trials and SMEs, a refined list of 16 factors across the four parameters (physical, psychological, environmental, and procedural) (Table 3) remained to holistically and objectively assess canine wellbeing. Veterinary consultations, by the nature, generally focus on physical health, which does not give a true representation of a dog's quality of life. Using the AWAG in practice means veterinary staff will discuss a dog's emotional state, its behavior and how it responds and copes with various aspects of its life. It will enable discussion of a dog's physical and social environment, and the predictability of its environment. The AWAG also encourages clinicians to consider the impact of the veterinary visit itself and the welfare concerns that may arise from treatment or procedures, or changes in housing/husbandry affecting the dog's environment which may not be considered in other welfare assessment tools. Owner decisions may be driven by emotion and focused on the potential outcome of treatment, they may not consider how various therapeutic methods may not be in the best interests of the dog (58), either in the long term or short term. Therefore, having the ability to quantify welfare and being able to show owners a visual representation of how their dog's welfare will score over time, may help as part of the decision-making process. Additionally, the AWAG is scored by the veterinary or animal welfare professional using the written descriptors, this helps mitigate any owner bias that may exist in other canine quality of life instruments.

Another utility of the AWAG is the ability to score and monitor the lifetime experience of a dog. Obtaining a baseline score as a puppy or when a dog is first seen in practice allows welfare to be tracked and gives insight into where interventions can be made to improve welfare. Regular monitoring provides clinicians and owners an objective overview of the key factors that influences dog wellbeing and encourages them to discuss psychological health, the environment, and veterinary and husbandry procedures that may be disregarded without use of the tool.

When designing the AWAG, there were also factors that the literature demonstrated could be indicators of welfare such as sleep and abnormal behaviors. Sleep has a significant relationship with mental and physical wellbeing in people. Humans who have depression experience changes in sleep; sleep continuity is affected as well as disinhibition of REM sleep (59–61). Quality and quantity of sleep and inactivity have recently been investigated as a measure of welfare in dogs. Inactivity is shown to be associated with anhedonia, depression-like states, and boredom (28). Conversely, inactivity may be indicative of relaxation or comfort, and it may be difficult to differentiate between inactivity as a result of distress vs. calmness, especially from owner reports. The ability to rest or sleep may also be inhibited by a stressful environment or may be extremely variable dependent on the dog's lifestyle and daily activity (62). Therefore, scoring sleep or inactivity on an ordinal scale from one to ten may also be unfeasible as the length of time a dog spends inactive, does not necessarily indicate a poorer emotional state (63) and quality of sleep is unlikely to be measured accurately without specialist equipment. The adaptability of the AWAG means in future research, wearables that monitor parameters such as activity, heart rate, sleep etc can be integrated into the AWAG site. These additional factors can give insight into a dog's wellbeing will provide additional data that allows animal welfare professionals to monitor of quality of life.

Abnormal behaviors in dogs can be indicative of negative affective states and poor welfare since they are commonly displayed in situations where an animal may be frustrated, stressed, fearful, or lacking stimulation and is often seen in environments where other indicators of poor welfare co-occur (51). These behaviors are out of context in terms of social or environmental stimuli or may be abnormal in duration, frequency and/or intensity, and they may be cognitively or emotionally damaging to the dog. Abnormal behavior can itself lead to welfare concerns if it causes physical injury such as self-mutilation. However, it also proved difficult to score abnormal behaviors on a scale. Denham et al. (64) found that abnormal behaviors can occur under a variety of conditions, not just in states of deprivation. They also found that stereotypic behaviors were reinforced by attention or another action the dog may find positive. Moreover, intensity of the behavior does not necessarily indicate poorer welfare and absence of these behaviors does not indicate good welfare. Therefore, it was decided not to include these factors as they would be problematic and give a potentially misleading assessment of dog wellbeing as in certain contexts or environments, abnormal behaviors may be helping the dog cope and may enhance welfare instead of decreasing it.

Having a valid and rapid tool for veterinary and animal welfare professionals to use is important to objectively assess welfare. Having confidence that an assessment tool has undergone a series of tests to evaluate if it accurately measures welfare and is reliable may reassure clinicians about their clinical judgement or help make treatment and management decisions.

One aim of this study was to provide initial validation of the AWAG for dogs and this was undertaken through both construct and content validity testing. This means that the tool would score a dog with a good quality of life with a low numerical score, and a dog in poor welfare would have a higher score. Veterinarians, veterinary nurses, canine welfare scientists, and clinical behaviorists are well-placed to judge if a dog is clinically and emotionally healthy in a stable environment. Therefore, these professionals were asked to score dogs that they deemed medically and emotionally healthy, dogs that were undergoing a procedure we know impacts welfare in the short-term (neutering), and dogs that have chronic medical or emotional problems. We found that healthy dogs scored low and dogs undergoing neutering scored significantly higher. Dogs with chronic conditions also scored significantly higher compared to healthy dogs. This demonstrates that the AWAG has the ability to accurately capture the welfare state of dogs and discriminate between varying stages of wellbeing. This is also seen in AWAG scores in other species (39, 41) where changes in both the CWAS and individual factor scores suggest subtle changes in welfare state and interventions can be made to improve welfare. Additionally, using the AWAG site, the user can see how their dog scores compared to the cohort of healthy dogs scored. If dogs score higher than the “average healthy dog”, this may encourage owners to make changes to improve their dog's wellbeing.

Another aim of this research was to assess the test re-rest reliability and the inter-rater reliability of the AWAG. The results indicate that when a healthy dog under non-changing conditions is assessed repeatedly over several weeks, there is very little variation between the scores. Several dogs had consistent scores throughout assessments, others had a variation of ~1, which is an expected finding as despite conditions remaining relatively stable, a dog's daily routine, environment and social interactions cannot be controlled completely, so their response and affective state will vary within and between each day.

Although there was no significant variation between scores when assessing inter-rater reliability, cumulative welfare assessment scores still showed some minimal variation, which could be because dogs may interact differently with different people, leading to different ratings. Moreover, veterinary clinics can be a stressful and rapidly changing environment for a dog, and their behavior and responses can change over a short period of time.

We believe the AWAG can be successfully utilized throughout the veterinary journey; in the consultation to discuss quality of life with the owner and identify where improvements can be made, during the hospitalization period to assess how the dog is coping in the environment, how they are impacted by procedural events, and during treatments to monitor if the dog's welfare is improving. Additionally, the AWAG can also be used to assess the welfare of dogs living in varying environments (rehoming shelters, assistance, and service dogs etc.) and allows the user to evaluate where interventions can be made to their environment and management events impact their quality of life.

## Future research

To provide further evidence of validity to the AWAG for dogs, assessing concurrent criterion validity would be of value, which would typically involve comparing a measure to another “gold-standard” measure; however, without a gold-standard measure against which the AWAG can be compared, other validated tools could be used to assess if the AWAG produces similar results, providing additional validity.

## Conclusions

This study reports the development of a novel canine welfare assessment tool that is highly accessible, produces instant results, easy to use, valid and reliable, and to be used by veterinary and dog welfare professionals with the aim of monitoring and improving dog quality of life. The AWAG for dogs provides a promising platform for clinicians to utilize to objectively quantify welfare to help measure the effects of interventions and to help make treatment and management decisions.

## Data availability statement

The original contributions presented in the study are included in the article/supplementary material, further inquiries can be directed to the corresponding author/s.

## Ethics statement

The animal study was reviewed and approved by University of Surrey NASPA, a Sub-committee of the Animal Welfare and Ethical Review Board (FHMS 20-21 182 EGA). Written informed consent was obtained from the owners for the participation of their animals in this study.

## Author contributions

RM data collection, analysis, and writing. SP and SW supervising, reviewing, and editing. All authors contributed to the article and approved the submitted version.

## Funding

The work was carried out with grants from Agria and SKK Research Fund.

## Conflict of interest

The authors declare that the research was conducted in the absence of any commercial or financial relationships that could be construed as a potential conflict of interest.

## Publisher's note

All claims expressed in this article are solely those of the authors and do not necessarily represent those of their affiliated organizations, or those of the publisher, the editors and the reviewers. Any product that may be evaluated in this article, or claim that may be made by its manufacturer, is not guaranteed or endorsed by the publisher.
